# A Systematic Review and Meta-Analysis of Ayurvedic Herbal Preparations for Hypercholesterolemia

**DOI:** 10.3390/medicina57060546

**Published:** 2021-05-28

**Authors:** Dinesh Gyawali, Rini Vohra, David Orme-Johnson, Sridharan Ramaratnam, Robert H. Schneider

**Affiliations:** 1 College of Integrative Medicine, Maharishi International University, Fairfield, IA 52557, USA; 2 School of Science of Consciousness, Maharishi University of Information Technology, Noida 201304, India; rinievohra@gmail.com; 3 Maharishi International University, Fairfield, IA 52557, USA; davidoj@earthlink.net; 4 Apollo Hospital, Greams Lane, Chennai, Tamil Nadu 600006, India; rsridharan52@gmail.com

**Keywords:** hypercholesterolemia, ayurveda, ayurvedic herbs, systematic review, meta-analysis

## Abstract

*Background and Objectives:* Cardiovascular disease (CVD) is the leading cause of death globally and hypercholesterolemia is one of the major risk factors associated with CVD. Due to a growing body of research on side effects and long-term impacts of conventional CVD treatments, focus is shifting towards exploring alternative treatment approaches such as Ayurveda. However, because of a lack of strong scientific evidence, the safety and efficacy profiles of such interventions have not been well established. The current study aims to conduct a systematic review and meta-analyses to explore the strength of evidence on efficacy and safety of Ayurvedic herbs for hypercholesterolemia. *Methods*: Literature searches were conducted using databases including Medline, Cochrane Database, AMED, Embase, AYUSH research portal, and many others. All randomized controlled trials on individuals with hypercholesterolemia using Ayurvedic herbs (alone or in combination) with an exposure period of ≥ 3 weeks were included, with primary outcomes being total cholesterol levels, adverse events, and other cardiovascular events. The search strategy was determined with the help of the Cochrane Metabolic and Endocrine Disorders Group. Two researchers assessed the risk of each study individually and discrepancies were resolved by consensus or consultation with a third researcher. Meta-analysis was conducted using the inverse variance method and results are presented as forest plots and data summary tables using Revman v5.3. *Results:* A systematic review of 32 studies with 1386 participants found randomized controlled trials of three Ayurvedic herbs, *Allium sativum* (garlic), *Commiphora mukul* (guggulu), and *Nigella sativa* (black cumin) on hypercholesterolemia that met inclusion criteria. The average duration of intervention was 12 weeks. Meta-analysis of the trials showed that guggulu reduced total cholesterol and low-density lipoprotein levels by 16.78 mg/dL (95% C.I. 13.96 to 2.61; *p*-value = 0.02) and 18.78 mg/dL (95% C.I. 34.07 to 3.48; *p* = 0.02), respectively. Garlic reduced LDL-C by 10.37 mg/dL (95% C.I. −17.58 to −3.16; *p*-value = 0.005). Black cumin lowered total cholesterol by 9.28 mg/dL (95% C.I. −17.36, to −1.19, *p*-value = 0.02). Reported adverse side effects were minimal. *Conclusion:* There is moderate to high level of evidence from randomized controlled trials that the Ayurvedic herbs guggulu, garlic, and black cumin are moderately effective for reducing hypercholesterolemia. In addition, minimal evidence was found for any side effects associated with these herbs, positioning them as safe adjuvants to conventional treatments.

## 1. Introduction

Cardiovascular disease (CVD) is the number one cause of death globally [1]. High blood pressure, high LDL cholesterol, and smoking are the key risk factors for CVD and about 49% of Americans have at least one of the three [2]. Hypercholesterolemia (hyperlipidemia/hyperlipoproteinemia/or dyslipidemia) is a condition characterized by an elevation of any or all parameters of lipid profile or lipoprotein levels in the blood [3]. Hypercholesterolemia generally means high levels of total cholesterol or LDL-C with normal or low levels of HDL-C. The guidelines of National Cholesterol Education Program (NCEP) Adult Treatment Panel III (ATP III) suggests LDL-C level < 100 mg/dL, (100–129) mg/dL, (130–159) mg/dL, and >160 as optimal, above optimal, borderline high, and high, respectively. It also suggests that the LDL-C should be the primary target of any cholesterol reducing therapy [4]. As with other types of CVD, genetics, age, and gender are some of the non-modifiable risk factors for hypercholesterolemia. Hypercholesterolemia is one of the most significant contributors to the development of CVD, and if managed properly, is directly responsible for reducing risk of morbidity and mortality associated with CVD [5,6] (The Lipid Research Clinics Coronary Primary Prevention Trial results I and II). The burden of hypercholesterolemia is also reflected by recurring acute cardiovascular events [7] and higher healthcare costs [8]. Hypercholesterolemia is one of the top 10 costliest medical conditions in 2008 in the US adult population [9].

### 1.1. Treatment Approaches

Diet and lifestyle modifications with or before starting the cholesterol-lowering drugs are the primary line of treatment for hypercholesterolemia [4]. Although emphasis is given to lifestyle modifications, a majority of people are required to take drugs to have an adequate reduction in LDL-C levels [10]. At present, the main drug class of choice for hypercholesterolemia is statins. Studies have suggested that statins can reduce the chance of heart attack and prevent consequent death by 30–40% and reduce LDL-C levels by 25–40%. Other alternatives to statins are fibrates, nicotinic acids, and cholesterol absorption inhibitors such as ezetimibe [4,10]. While these cholesterol-reducing drugs are generally considered safe, they are not free from side effects [10]. It is well established now that statin use is highly associated with adverse events and their manifestations such as myositis, myalgia, rhabdomyolysis, cognitive loss, neuropathy, pancreatic and hepatic dysfunction, and sexual dysfunction [11]. In fact, statin use is also known to increase the risk of new-onset diabetes from anywhere between 28–43% [12]. Many times, drugs such as statins and ezetimibe do not even reach the desired reduction in LDL levels, with residual CVD risk still persisting [13]. Adverse events associated with conventional treatments is also one of the reasons why a large percentage of the US population does not treat hypercholesterolemia despite being aware of the condition [14]. Such issues have prompted researchers to explore alternative/integrative treatment approaches and multiple new therapies are emerging in that area [13]. Complementary and alternative medicine (CAM) treatments have shown significant benefits among individuals with hypercholesterolemia [15]. One of the CAM therapies that has shown promising results for hypercholesterolemia is Ayurveda [16]. 

### 1.2. Ayurveda

Ayurveda (translated as “the science of life”) is one of the oldest medical systems in the world. Its origins date back to thousands of years ago in the Vedic era in the Indian subcontinent. Ayurveda defines life, “ayu”, as a union of mind, body, spirit, and senses and health as the balanced state of these factors [17]. The wisdom of Ayurveda is based on three major classical texts, namely Charaka Samhita, Sushruta Samhita, and Ashtanga Hridaya, plus six minor texts. These ancient texts give detailed descriptions of over 700 herbs and 6000 formulations in addition to descriptions of various diseases, diagnostic methods, and dietary and lifestyle recommendations [18]. Ayurvedic treatment focuses on restoring the balance of the disturbed body–mind matrix through diet and behavioral modifications, administration of drugs, and detoxification and rejuvenation therapies. The branch of Ayurvedic science that deals with herbs and their qualities is called Dravyaguna vigyan. Ayurvedic formulations are prepared based on this knowledge and largely comprise herbs. Classical and proprietary Ayurvedic formulations may consist of a single herb or mixtures of many herbs in any form, viz., juice, extract, powder, tablet, or decoction.

Although there is no direct correlate for hypercholesterolemia in Ayurveda, dyslipidemia can be considered close to the Ayurvedic terms “medovriddhi” or “medodushti”. The main herbs used in Ayurveda to reduce cholesterol are garlic (*Allium sativum*), guggulu (*Commiphora mukul*), and arjuna (*Terminalia arjuna*) [19,20,21]. The authors of this paper looked into the most common Ayurvedic products used for high cholesterol. Either used alone or in combination with other herbs, these three herbs are found in most of the Ayurvedic formulations with some additional ingredients. The list of additional ingredients used in combination with the above-mentioned herbs may include pushkarmoola (*Inula racemosa*), ginger, turmeric, shilajit, punarnawa (*Boerrhavia diffusa*), triphala, *Nigella Sativa*, garcinia, *Cyperus rotundus,* and licorice. Many published clinical trials on Ayurvedic herbs for hypercholesterolemia have presented some evidence that these formulations are effective in reducing cholesterol [19,20,21]. However, many times, such RCTs are often limited by their study designs, sample sizes, or lack of validity and/or generalizability [22]. Recently, researchers have also been encouraged to apply principles of evidence-based medicine to Ayurveda [17,23]. 

### 1.3. Need for Study

Although there are many reviews for individual Ayurvedic herbs [20,24,25,26,27], there is a strong need to conduct a review to systematically summarize the available evidence as well as identify the strength of this evidence. Our preliminary search yielded one systematic review on the use of Ayurvedic herbs for Hyperlipidemia. Singh et al. (2007) [16] conducted a systematic review on Ayurvedic herbs and collateral treatments for hyperlipidemia and concluded that a significant number of researches show strong efficacy of Ayurvedic herbs for hyperlipidemia, with minimal reports of side effects. Despite its comprehensiveness, the review was limited by the use of randomized and quasi-randomized studies, arbitrary scoring methods to categorize studies, and a lack of systematic summarization of results using meta-analyses. With the current study, we aim to critically analyze the available evidence on potential benefits and harms of Ayurvedic herbs for hypercholesterolemia using Cochrane guidelines for conducting systematic reviews and meta-analyses. The current study adds on the systematic review of Singh et al. (2007) by providing strong conclusions using statistically accurate methods to establish unambiguous evidence. 

## 2. Materials and Methods 

The current review was conducted in accordance with the Preferred Reporting Items for Systematic Reviews and Meta-Analyses (PRISMA) guidelines [28], and a protocol was previously published with the Cochrane database [29] for systematic reviews and meta-analysis [30]. 

### 2.1. Search Strategy

A systematic literature review of all studies published and accessible through December 2020 was performed by two authors (DG and RS) using the following databases:

- The Cochrane Library, Cochrane Database of Systematic Reviews (CDSR), Cochrane Controlled Trials Register (CENTRAL), Database of Abstracts of Reviews of Effectiveness (DARE), Health Technology Assessment Database (HTA), MEDLINE, EMBASE, AMED (Allied and Complementary Medicine Database), World Health Organization (WHO) ICTRP (International Clinical Trials Registry Platform-http://apps.who.int/trialsearch/, accessed on 1 December 2020), ClinicalTrials.gov, EU Clinical Trials Register, and Europe PubMed Central. A MEDLINE (via Ovid platform) email alert service was continuously applied to identify newly published studies using the same search strategy as described for MEDLINE. If any additional relevant key words were detected during any of the electronic or other searches, the electronic search strategies were modified to incorporate these terms and document the changes.
- Clinical Trial Registry India, AYUSH research portal (Evidence Based Research Data of AYUSH Systems at Global Level, Department of AYUSH, Ministry of Health & Family Welfare, Government of India), *Journal of Research in Ayurveda and Siddha, The Journal of Research & Education in Indian Medicine* (JERIM), *AYU* (publication of Gujarat Ayurveda University, India), *The International Journal for Ayurveda Research, Journal of Drug Research in Ayurveda, Journal of Ayurveda and Integrative Medicine*, *Ancient Science of Life*, *International Journal of Ayurveda and Pharma Research*, A Bibliography of Indian Medicine (ABIM), Digital Helpline for Ayurveda Research Articles (DHARA), *Indian Heart Journal*.
- Other resources. Every effort was made to identify other potentially eligible trials or ancillary publications by searching the reference lists of retrieved included trials, systematic reviews, meta-analyses, and health technology assessment reports. In addition, study authors of included trials were contacted to identify any further studies that may have been missed.

Selection of studies: Abstract, title, or both of every record retrieved was scanned to determine which studies should be assessed further. All potentially relevant articles were investigated as full text. In case of any discrepancy, consensus was made with a discussion between all authors. An adapted PRISMA (Preferred Reporting Items for Systematic Reviews and Meta-Analyses) flow diagram was presented showing the process of study selection [28]. For studies fulfilling inclusion criteria, key participant and intervention characteristics were abstracted and data on efficacy outcomes and adverse events were reported using standard data extraction templates as supplied by the Cochrane Metabolic and Endocrine Disorders Group and Cochrane Hypertension Group. Efforts were made to find the protocol of each included study, and primary, secondary, and other outcomes are reported in comparison with data in publications in a joint appendix, “Matrix of study endpoint (publications and trial documents)”. Duplicate studies, companion documents or multiple reports of a primary study, and yield of information was maximized by collating all available data, and the most complete dataset aggregated across all known publications was used. In case of doubt, priority was given to the publication reporting the longest follow-up associated with primary or secondary outcomes of these studies. 

Types of studies: All relevant randomized controlled trials (RCTs) irrespective of publication status, blinding, and language were included. The original authors were contacted to confirm the details on random list generation and allocation concealment when possible. Quasi-randomized or non-randomized and studies shorter than 3 weeks in duration were not included. However, those studies were separately analyzed to document the available evidence. Trials that studied non-pharmacological approaches of Ayurveda (for example, Panchakarma) as a single intervention were excluded. Where participants were given some other treatments such as statins, in addition to Ayurvedic herbal preparations, the studies were included if the treatment was evenly distributed between groups and it was only Ayurvedic treatment that was randomized.

Participants: All studies where participants have high blood cholesterol levels (diagnosed as per the standard laboratory tools) without restrictions of age, gender, ethnicity, and other medical conditions were included. Study participants were considered eligible irrespective of the duration and chronicity of the condition and/or treatment duration. Studies with participants having a mean total cholesterol level greater than 200 mg/dL (5.2 mmol/L) or LDL cholesterol > 130 mg/dL were included. ATP III suggests above readings are the levels of borderline high risk (NCEP, 2001). Studies where participants are not subject to standard laboratory tests to diagnose hypercholesterolemia were not included.

Interventions: The following comparisons of intervention versus control/comparator were carried out.

(a) Ayurvedic herbal preparations. These include extracts from mixtures of herbs, single herbs, Ayurvedic proprietary medicines, or a compound of herbs that are prescribed by an Ayurvedic practitioner. All the available interventions under this category, regardless of their mechanism of action, were included;
(b) Ayurvedic herbal preparations in addition to standard care. Studies with Ayurvedic herbal medicines and conventional treatment for cholesterol (for example statins) as an intervention were also included as long as both the arms of the randomized trials received the conventional treatment.

#### 2.1.1. Comparison Groups

- Placebo compared with (a) or (b);
- Usual care compared with (a) or (b);
- Non-pharmacological intervention (for example diet, exercise, or both);
- No intervention.

#### 2.1.2. Outcomes

##### Primary Outcomes

- Total cholesterol levels;
- Adverse events;
- Major adverse cardiovascular events such as MI, stroke.

##### Secondary Outcomes

- Serum triglyceride levels;
- High-density lipoprotein (HDL) levels;
- Low-density lipoprotein (LDL) levels;
- Changes in body mass index (BMI) and body weight;
- Morbidity and or mortality;
- Health-related quality of life;
- Socioeconomic effects.

Method and timing of outcome measurement: A systematic method was applied to measure outcomes both method wise and timing wise. Regarding methods, standardized measurement instruments were used for those outcomes which can be measured objectively, such as lipid levels, BMI, and body weight. For other outcome measures such as health-related quality of life and socioeconomic effects, standardized/valid scales of measurements were used when available, or widely acceptable definitions of the outcomes were followed.

All the studies were categorized in two broad groups based on timing of outcome measurement “short-term” group with timing of at least 3 weeks and “long-term” group of more than 6 months. Outcomes including lipid levels, BMI, and body weight were considered in the short-term group whereas other outcomes such as health-related quality of life, adverse events, morbidity, and mortality, along with lipid levels, BMI, and body weight, were considered in the long-term group.

Data collection, analysis, and calculation of treatment effects: We used pre-designed standard data abstraction forms to collect data and software Revman, Version 5.3 (The Cochrane Collaboration, The Nordic Cochrane Centre, Copenhagen, Denmark) to enter, analyze, and synthesize the data. Results were presented in forest plots and data summary tables. Dichotomous data were calculated in risk ratio or odds ratio with a 95% confidence interval (CI) whereas the continuous data were calculated in mean difference with a 95% CI and standardized mean difference with a 95% CI. 

Meta-analysis method: Inverse variance method was used for meta-analysis. Dichotomous data were expressed as odds ratios (ORs) or risk ratios (RRs) with 95% confidence intervals (CIs). Continuous data were expressed as mean differences (MDs) with 95% CIs. Time-to-event data were expressed as hazard ratios (HRs) with 95% CIs. The formulas for meta-analysis were used as described by Deeks and Higgins in the supplementary statistical guidelines for the software Revman 5.3 [31]. The level at which randomization occurred was closely monitored, such as cross-over trials, cluster-randomized trials, and multiple observations for the same outcome.

#### 2.1.3. Assessment of Risk of Bias in Included Studies 

DG and RS assessed risk in individual studies independently. Disagreements were resolved by consensus, or by consultation with SR. The Cochrane Collaboration’s tool was used to assess the risk of bias [29,32]. The following criteria were assessed for this purpose:

- Random sequence generation (selection bias);
- Allocation concealment (selection bias);
- Blinding of participants and personnel (performance bias);
- Blinding of outcome assessment (detection bias);
- Incomplete outcome data (attrition bias).

Risk of bias criteria was judged as “low risk”, “high risk”, or “unclear risk” and individual bias items were evaluated as described in the *Cochrane Handbook for Systematic Reviews of Interventions* [29].

Missing Data: Missing data were obtained from authors where possible, and reasons for missing data (attrition rates, e.g., drop-outs, losses to follow-up and withdrawals, issues of missing data and imputation methods (e.g., last observation carried forward [LOCF])) were investigated and critically appraised. Missing standard deviations (SD) were imputed (average of SD of studies where reported) and the impact of imputation on meta-analyses was investigated by sensitivity analyses. 

Assessment of Heterogeneity: Causes of any significant clinical, methodological, or statistical heterogeneity were explored but the pooled effect estimate in a meta-analysis was still presented. Heterogeneity was identified through visual inspection of the forest plots and by using a standard chi-square test ⍺ and the I² statistic < 75% [33]. If 10 or more studies were included investigating a particular outcome, funnel plots were used to assess small study effects. Several explanations can be offered for the asymmetry of a funnel plot, including true heterogeneity of effect with respect to trial size, poor methodological design (and hence bias of small trials), and publication bias. Therefore, results were interpreted carefully [34].

#### 2.1.4. Data Analyses

Revman (Version 5.3) was used to compute effect sizes as well as other statistical information such as *p*-values, t-scores, Q statistics, and confidence intervals. Forest plots, funnel plots, and data summary tables were created utilizing this software. Unless there was good evidence for homogeneous effects across studies, primarily low risk of bias data was summarized using a random-effects model [35]. Random-effects meta-analyses were interpreted with due consideration of the whole distribution of effects by presenting a prediction interval [36]. A prediction interval specifies a predicted range for the true treatment effect in an individual study [37]. Statistical analyses were performed according to the statistical guidelines in the latest version of the *Cochrane Handbook for Systematic Reviews of Interventions* [29].

### 2.2. Subgroup Analysis and Investigation of Heterogeneity

The following characteristics were expected to introduce clinical heterogeneity and, when possible, subgroup analyses were conducted:

- Age;
- Ethnicity;
- Geographical location;
- Diet pattern (Indian diet and Western diet, salt-restricted diet and salt-unrestricted diet, etc.)

### 2.3. Sensitivity Analysis

Sensitivity analyses was performed in order to explore the influence of the following factors (when applicable) on effect sizes:

- Restricting the analysis to published studies;
- Restricting the analysis by considering risk of bias, as specified in Section 2.1.3 (Assessment of Risk of Bias in Included Studies);
- Restricting the analysis to very long or large studies to establish the extent to which they dominate the results;
- Restricting the analysis to studies using the following filters: diagnostic criteria, imputation, language of publication, source of funding (industry versus other), and country.

The robustness of the results was tested by repeating the analysis using different measures of effect size (RR, odds ratio (OR), etc.) and different statistical models (fixed-effect and random-effects models).

### 2.4. Including Non-Randomized Studies

When there were only a small number of randomized studies identified for systematic review and meta-analysis, non-randomized studies were also included. These non-randomized studies may be quasi-randomized, controlled clinical trials, or simply before-after clinical trials.

However, data from both randomized and non-randomized studies were not combined together in the same analysis as this may affect the strength of the evidence. The guidelines from *Cochrane Handbook of Systematic Reviews and Meta-Analysis* says that “where randomized trial evidence is desired but unlikely to be available, eligibility criteria could only be structured to say that nonrandomized studies would only be included where randomized trials are found not to be available. In time, as such a review is updated the non-randomized studies may be dropped when randomized trials become available” [29] (p. 397).

## 3. Results

**Figure 1:**
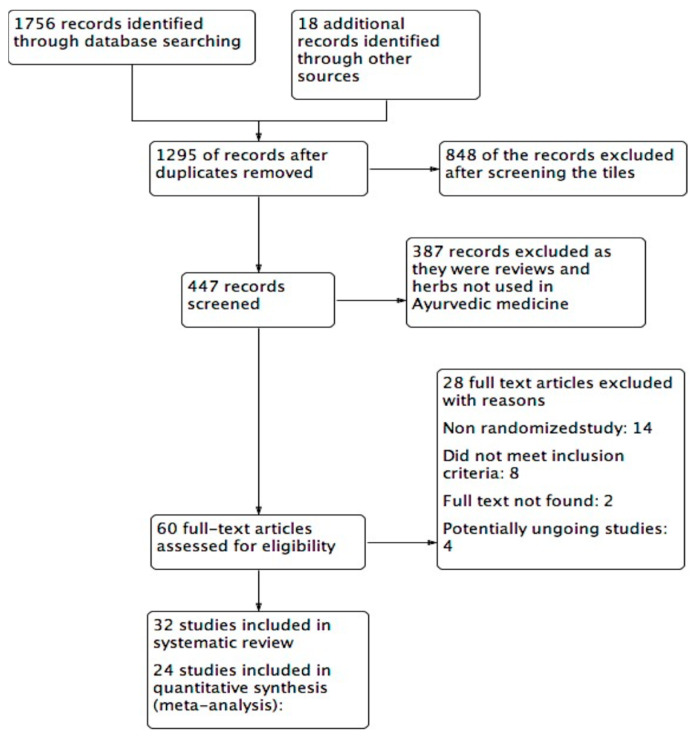
Study flow diagram on Ayurvedic herbal preparations for hypercholesterolemia.

A total of 1756 potentially relevant studies were found by searching the databases MEDLINE, CENTRAL, AMED, EMBASE, WHO ICTRP, Dhara online, AYUSH research portal, Clinicaltrials.gov, and INDMED. Through hand searches, 18 more studies were identified. After duplication and screening of the titles of obtained records, a total of 447 studies were considered for further screening. After perusal of the titles and abstracts, 387 studies were excluded due to the following reasons: focusing on herbs not part of Ayurveda (Western and Chinese herbs), reviews, observational studies, and not meeting the inclusion criteria. Sixty studies were found potentially eligible at this stage and the full papers were obtained. Among these, 14 studies were non-randomized, 8 studies did not fulfill initial inclusion criteria, 4 were either incomplete or potentially ongoing, and the full text of 2 studies could not be obtained. Hence, only 32 studies were ultimately included in the systematic review, of which 24 studies were qualified to be included in the meta-analysis. The characteristics of the included studies are included in Table 1. An adapted PRISMA [28] flow-chart of the study selection appears in Figure 1.

Excluded studies: Among potentially relevant studies, 28 were excluded for the following reasons. Two studies were of short duration, fourteen were non-randomized, and two could not be included as their full text could not be retrieved. Ten studies did not meet the minimum inclusion criteria. Studies investigating the use of Western herbal preparations that are not used in Ayurveda and pharmacological studies were excluded. Studies of garlic using garlic oil or aged garlic were also excluded because these are not described in classical Ayurvedic literature.

Risk of bias assessment: Although the majority of the studies were randomized and double-blind, they failed to provide details of random sequence generation and allocation concealment. It was definitely inadequate in the majority of the studies. Only six studies out of 32 explained the random sequence generation. Two studies had high risk of bias in blinding whereas seven studies did not specify their blinding status. Both participants and investigators were blinded in 22 studies. In general, the blinding was achieved by using identical looking treatment and placebo tablets or capsules. However, the blinding of participants and investigators was more common than the blinding of outcome assessors. There was less information about the outcome assessment methods and personnel. Fifteen studies were prone to have attrition bias as the dropouts were not included in the final analysis. In one of the studies [59], 50% withdrawal was reported and the explanation was given as “unavoidable circumstances”. In another study [48], 39 out of 64 participants in the treatment group and 34 out of 59 participants in the control group completed the study. Since many of the studies did not provide information on their protocol, it is hard to say if selective outcome reporting bias existed. However, based on the methods in the studies, all but three of the studies did not seem to have selective outcome reporting bias. A table of risk of bias assessment is given in Table 2.

### 3.1. Effects of Ayurvedic Herbs 

#### 3.1.1. Total Cholesterol (TC) (mg/dL)

Overall, Ayurvedic herbal formulations were found to be effective in reducing total cholesterol by approximately 7.5%. A meta-analysis of 24 randomized controlled trials on four different Ayurvedic interventions, namely garlic, guggulu, *Nigella sativa,* and a combination of garlic and guggulu (*Lashunadi Guggulu*), involved a total of 1386 participants with 699 in the Ayurvedic group and 687 participants in the control group (Table 3).

**Figure 2:**
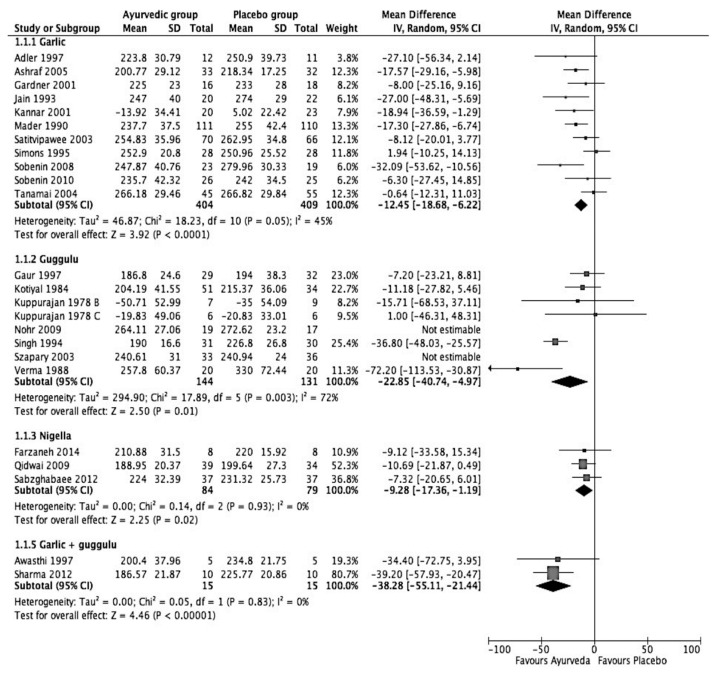
Forest plot on effect of Ayurvedic herbal preparations on total cholesterol (mg/dL).

Figure 2, a forest plot of the meta-analysis, shows that the most effective intervention is Lashunadi Guggulu (garlic + guggulu), with a reduction of 38.28 mg/dL in TC (95% C.I.: −55.11 to 21.14; *p* < 0.00001). The second most effective intervention was guggulu (*Commiphora mukul*), reducing TC by 16.78 mg/dL (95% C.I.: −13.96 to −2.61; *p* = 0.02) or almost 8.5% of borderline high TC levels. The third most effective intervention for reducing high TC was found to be garlic. Analysis of findings from 11 studies comparing 404 participants taking garlic with 409 participants on a placebo showed that garlic reduces TC by 12.45 mg/dL (95% C.I.: −18.68 to −6.22, *p* < 00001). Finally, the intervention with the least effect was found to be *Nigella sativa,* reducing TC by 9.28 mg/dL (95% C.I.: −17.36 to −1.19; *p* = 0.02). 

#### 3.1.2. LDL Cholesterol (mg/dL)

**Figure 3:**
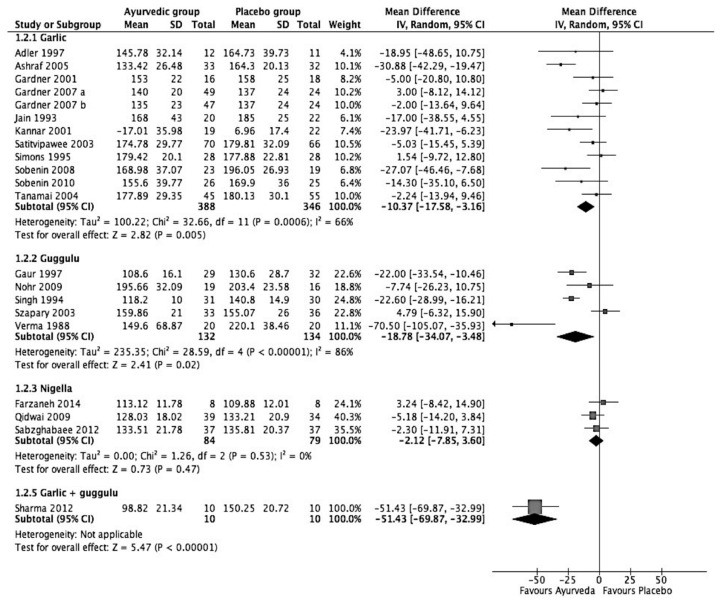
Forest plot on the effect of Ayurvedic herbal preparations on LDL-C (mg/dL).

As shown in Table 4 and Figure 3, garlic was found to reduce LDL-C by 10.37 mg/dL (95% C.I.: −17.58 to −3.16; *p* = 0.005). This result is nearly 8% of the borderline LDL-C levels. The heterogeneity between garlic studies was 66%. As compared to the placebo, guggulu was found to reduce LDL-C by −18.78 mg/dL (95% C.I.: −34.07 to −3.48; *p* = 0.02). Unlike the results for total cholesterol, *Nigella sativa* did not have significant effects on a reduction in LDL-C (2.12 mg/dL (95% C.I.: −7.85 to 3.6; *p* = 0.47)), as shown in Figure 3. Altogether, these studies included 163 participants, of which 84 people were in an intervention group and the remaining 79 were in the control group.

#### 3.1.3. Triglycerides (mg/dL)

**Figure 4:**
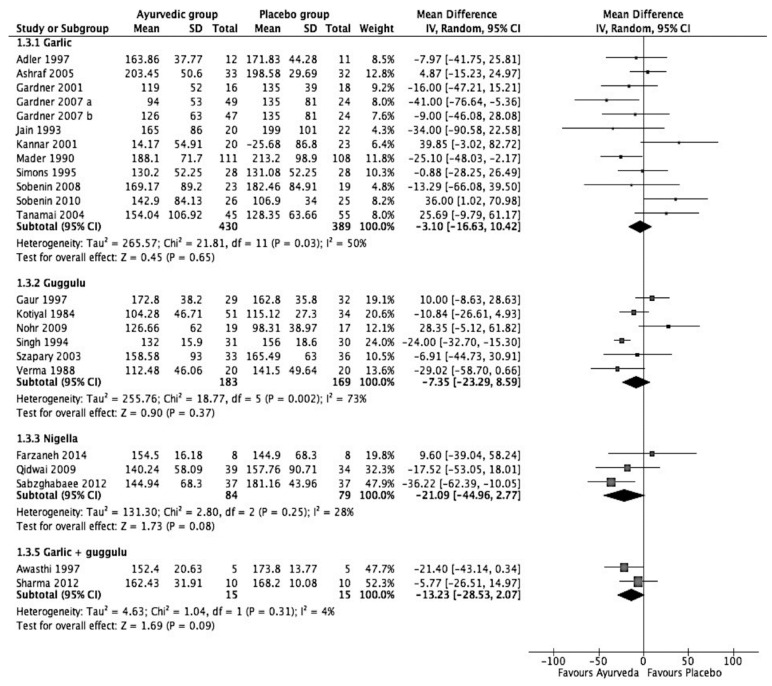
Forest plot on effect of Ayurvedic herbal preparations in triglycerides (mg/dL).

Meta-analyses of the four interventions showed (Table 5 and Figure 4) garlic to be the least effective in reducing raised TG levels (3.1 mg/dL (95% C.I.: −16.63 to 10.42; *p* value = 0.65)), as shown in Figure 4. *N. Sativa* was found to be the most effective intervention, where the meta-analysis of three studies showed that it reduces TG by −21.09 mg/dL (95% C.I.: −44.96 to −2.77; *p* value = 0.08). Although the confidence interval of the effect size is wide and the *p* value of the final effect is 0.08, the heterogeneity among the studies was fairly low at 28%. *Lashunadi guggulu*, according to the combined results of two small studies, reduces TG levels by 13.23 mg/dL (95% C.I.: −28.53 to 2.07; *p* value = 0.09). Six studies on guggulu, when meta-analyzed, showed that guggulu helps to reduce TG levels by 7.35 mg/dL (95% C.I.: −23.29 to 8.59; *p* value = 0.0.37). Here, two studies that showed positive results in other cholesterol levels are negative and opposite in one study.

#### 3.1.4. HDL (mg/dL)

**Figure 5:**
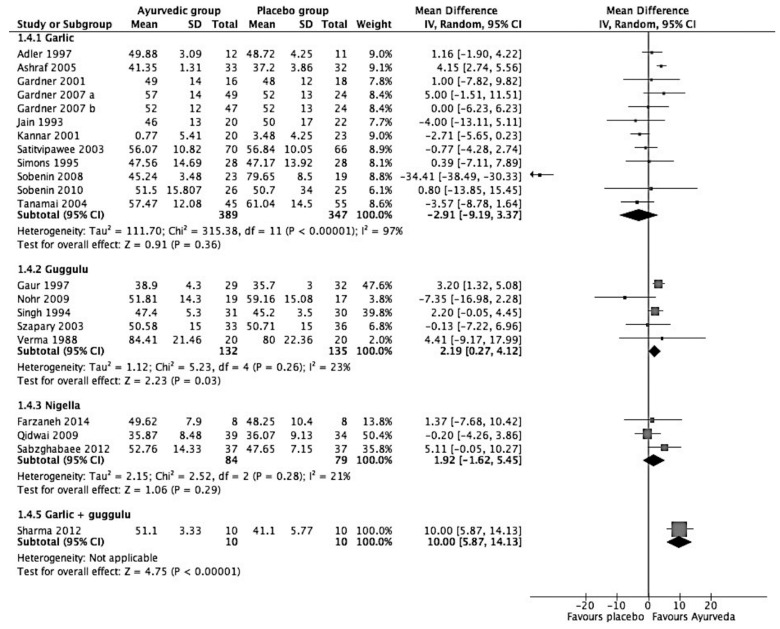
Forest plot on effect of Ayurvedic herbal preparations in HDL-C (mg/dL).

The meta-analysis of 21 RCTs with 1186 participants (615 in the Ayurvedic group and 571 in the placebo group, as shown in Table 6) suggest the statistically non-significant effect of Ayurvedic interventions on HDL-C. Guggulu alone and when mixed with garlic, however, showed positive and statistically significant results in increasing HDL-C. Analysis of end results from five RCTs in guggulu involving 264 total subjects showed that, as compared to the placebo, guggulu increased HDL-C by a small but significant difference of 2.19 mg/dL (95% C.I.: 0.27 to 4.12; *p* value = 0.03). On the other hand, results from a single study showed that *Lashunadi guggulu* was found to be raising HDL-C levels by 10 mg/dL (95% C.I.: 5.87 to 14.13; *p* < 0.00001). *N. sativa* also did not seem to have a significant effect on HDL-C. Results of the meta-analysis of three studies showed that it raised HDL-C levels by 1.92 mg/dL (95% C.I.: −1.62 to 5.45; *p* = 0.29). These studies involved a total of 163 participants. Garlic was also found to have no significant effect on HDL-C levels. Among 12 studies involving 736 participants, five studies claimed that garlic reduces HDL-C, and one study by Gardner et al. [51] found out that garlic neither reduces nor increases HDL-C. The studies were also highly heterogeneous with a high 97% I^2^ statistic. Though it may not even be relevant to conduct a meta-analysis on the effects of garlic on HDL-C, it is presented in the forest plot to show the current evidence (Figure 5). 

## 4. Discussion

The findings of the current meta-analyses are consistent with the clinical experiences and recommendations of traditional Ayurvedic literature. The two promising herbs for clinical improvements in hypercholesterolemia were found to be garlic and guggulu. This systematic review and meta-analysis suggested that Ayurvedic herbal preparations are safe and effective in reducing major cholesterol biomarkers. In addition to the studies conducted on these four interventions, 32 randomized controlled studies found out that there are 10 additional Ayurvedic interventions such as holy basil, ginger, fenugreek, and Indian gooseberry, which are capable of correcting hypercholesterolemia. However, a majority of studies were conducted on garlic, guggulu, *Nigella sativa,* and *Lashunadi guggulu* (a garlic and guggulu combination). It was also observed that amongst all of these Ayurvedic interventions, garlic and guggulu stood out as the most effective interventions. Although *Lashunadi guggulu* topped the list in its effectiveness, due to the lack of enough number of studies, the strength of the evidence on this finding is fairly low. Garlic and guggulu had the most consistent effects on cholesterol, except garlic did not seem to be as effective in increasing HDL-C as compared to guggulu. Both garlic and guggulu were not found to be effective in reducing TG levels, as the effect size was not statistically significant. Studies on garlic were less heterogeneous than studies conducted on guggulu. However, the overall effect size of guggulu exceeds that of garlic. 

### 4.1. Commiphora Mukul (Guggulu)

Amongst all of the Ayurvedic interventions studied, *Commiphora mukul*, commonly known as guggul(u) was found to have the biggest effect size. Seven randomized controlled trials in eight trial arms enrolling a total of 380 participants compared guggulu with a placebo for its effect on various cholesterol levels. It was observed that guggulu reduces TC by 16.78 mg/dL (95% C.I.: 30.96 to 2.61, *p* value = 0.02) and LDL-C by 18.78 mg/dL (95% C.I.: 34.07 to 3.48, *p* value = 0.02). These findings on guggulu came from the analysis of end results from eight trial arms of seven RCTs involving 380 people (184 in the control group and 197 in the experimental group). One of the studies included in this analysis [66] seems to have had a big influence on the overall effect of this intervention. Heterogeneity among these seven studies was 75%, which still allowed the conduction of the meta-analysis. Likewise, as compared to a placebo, guggulu reduced TG levels by 7.35 mg/dL (95% C.I.: 23.29 to 8.59; *p* value = 0.37) and raised HDL-C by 2.19 mg/dL (95% C.I. 0.27 to 4.12; *p*-value = 0.03). This effect size counts for a reduction in TC and LDL-C by nearly 6.5% and 10%, respectively. This finding is of clinical significance, as it is associated with a 38% reduction in the risk of coronary events at age 50 (Law et. al, 1994) [69]. The risk of coronary events is *dependent* on other cardiovascular risk factors such as hypertension and age. In addition, it is also understood that a 10% reduction in LDL-C levels helps minimize the risk of coronary and vascular events [70]. Out of eight trial arms included in analysis, all but two studies [21,47] were conducted in the Western population with a typical Western diet. When a sensitivity analysis was performed by excluding these two studies, then the effect size was 22.85 (95% C.I.: 40.74, 4.97). Another possibility is that the discrepancies are due to the dietary habits of Indian and Western populations. Another postulation could be that this native Indian plant is better suited to natives of India—as the Vedic scriptures say, “local plants and herbs are best suited to the local people” [71]. 

One of the studies included in the analysis of guggulu is Verma (1988) [66] and shows a bigger effect size. However, this study included patients with a baseline total cholesterol level of 275 mg/dL or more or triglycerides levels of 200 mg/dL. Thus, there is a possibility that guggulu is more effective when the baseline cholesterol and triglyceride levels are high. Moreover, Verma (1988) describes the process of purification of guggulu. Purification of guggulu is also associated with its chances of posing any side effects. Although there were no serious adverse events posed by guggulu, a small proportion of participants did experience diarrhea, headaches, and skin rashes. These side effects are minimal when compared to the threats posed by conventional drug therapy. Purified guggulu seems to have fewer side effects. The study by Szapary et al. [21], which showed negative effects of guggulu, did not mention if the product was purified as per the Ayurvedic protocol or not. Additionally, the study included an extracted version of guggulu prepared by a commercial manufacturer. Thus, there is an inherent chance of bias despite the fact that it was the first methodologically sound randomized controlled trial conducted on guggulu in the West. When asked for further comments, no response was received from the principal author [21]. 

So far, the types of guggulu used for clinical trials are very diverse. Some studies used purified crude gum guggulu as recommended by Ayurvedic texts, whereas others used a guggulu extract called guggulipid. Guggulipid is extracted from the plant by using ethyl acetate and is mixed with petroleum ether to produce a product called fraction A [26]. This fraction A guggulu has been used in some trials. Extracting the active ingredient of guggulu is not an Ayurvedic practice, so it might be a plausible reason for this observation. Guggulu has been qualitatively reviewed by few several groups of researchers in the past [16,26,72]. So far, none of the reviews conducted a meta-analysis of guggulu studies. This work is first of its kind and thus cannot be compared with any previous meta-analysis. In summary, guggulu is moderately effective in terms of total cholesterol, LDL-C, and HDL-C, and there is a strong evidence to this end. Thus, it can be recommended as an adjuvant to cholesterol-lowering pharmacological therapy or as a supplement to a healthier diet and lifestyle for those who have borderline cholesterol levels. 

#### 4.1.1. Garlic

It was observed that garlic reduces total cholesterol by almost 5% and LDL-C by 6% in subjects with elevated total cholesterol levels (mean TC > 200 mg/dL), and has a statistically non-significant effect on HDL-C and triglycerides. This observed reduction in LDL and total cholesterol is clinically relevant as it is associated with a reduction in the risk of adverse coronary and vascular events [69,70]. 

The results also suggested that garlic was highly tolerable and does not pose any side effects, which are more common with conventional therapies. In a majority of the studies, bad smell or odor was the only major side effect of garlic. A small proportion of the population did experience some gastrointestinal issues such as belching and acid reflux, but in comparison to statins, these side effects are not serious. Statins, on the other hand, do pose some serious adverse effects including a high risk of diabetes, cognitive and muscular impairment, sexual dysfunction, mood swings, anxiety, and irritability [73]. 

These findings corroborate the results of previous meta-analyses and systematic reviews [74,75,76]. In previous literature, the effect of garlic on cholesterol levels has been a debatable topic. Time and again, many individual trials of garlic have reported diverse therapeutic effect size of garlic on cholesterol levels. The study by Stevinson et al. [76] was one of the earliest meta-analysis of garlic on hypercholesterolemia and included 13 trials, whereas the recent and most updated study, by Ried et al. [74], included 39 studies. The studies by Stevinson et al. and Ried et al. both suggest that garlic reduces TC levels by approximately 15 mg/dL, whereas the study by Reinhart et al. [75] suggests its effect size is nearly half of what was observed by previous studies, i.e., −7.34 mg/dL. They attribute this smaller effect size to including newer studies, which exhibit more modest effects than older studies. However, the recent and most updated meta-analysis on garlic by Ried et al. [74] seems to be the most comprehensive one. 

Meanwhile, observed results from this study have suggested that garlic has a modest effect size of −12.45 mg/dL. One reason behind this finding may have to do with the type of included studies in this review. Because Ayurveda does not entertain the use of aged garlic, extracted garlic oil, or extracted compound of allicin from garlic, and uses whole garlic as a preparation, this work did not include any of the studies that used those various forms of garlic as an intervention. There is a common trend of using aged garlic and a lot of studies had to be excluded for using aged garlic as an intervention. However, the studies that used whole garlic or dried garlic powder were included, as it is the common practice to use whole garlic cloves in Ayurvedic pharmaceutical science [71]. From a pharmacological point of view, it is believed that the active compound called allicin, a garlic derivative, is responsible for reducing cholesterol levels and for the distinctive smell of garlic [77]. Allicin is a volatile compound and is responsible transiently for cardiovascular effects [77]. However, the exact ingredients and their mechanism of action still remains unknown. On the other hand, Ayurveda has been using garlic for treating Hridroga, an Ayurvedic term for cardiovascular diseases [71]. In Ayurveda, garlic, which possesses all five tastes except sour taste, is capable for clearing channels and all the coverings (avarana) because of its pungent and piercing qualities [71,78]. 

In conclusion, the findings of this review suggest that garlic is superior to placebos in reducing elevated total cholesterol and LDL-C levels. Garlic has also been shown to have additional cardiovascular benefits such as reducing high blood pressure [79]. When taken together, garlic preparations can be considered as a general heart tonic with cholesterol regulating properties. It can also be used as a preventive agent in borderline cholesterol levels, with a higher safety and tolerability profile than statins. 

#### 4.1.2. Nigella Sativa

Another intervention that showed some efficacy on cholesterol levels was *Nigella sativa*. Commonly known as black cumin and Upakunchika in Ayurveda, it is more famous for its digestive effect. Ideally, *Nigella sativa* seed powder is used in Ayurveda, so studies of its seed oil or other extracts were not included. Based on the analysis of three studies on *Nigella sativa*, it was observed that it reduces total cholesterol by 9.28 mg/dL (95% C.I.: 17.36 to 1.19, *p* = 0.02). However, it does not seem to have a statistically significant effect on other parameters such as LDL-C 2.12 mg/dL (95% C.I.: 7.85 to 3.6; *p* = 0.47), triglycerides 21.09 mg/dL (95% C.I.: to −2.77; *p* = 0.08), and HDL-C 1.92 mg/dL (95% C.I.:1.62 to 5.45; *p* = 0.29). This observation is comparable to the results of a recently published systematic review by Sahebkar et al. [80]. The difference between the current study and [80] is the inclusion criteria; the present study only included studies with whole seed powder, whereas Sahebkar et al. [80] included other versions of *Nigella sativa* such as seed oil and also had no restriction on baseline total cholesterol levels for the inclusion criteria, which limited the number of studies in this meta-analysis.

As an addition to the herbs mentioned above, we also conducted a systematic review to examine the effects of *Terminalia arjuna* on lipid parameters of hypercholesterolemic patients. Due to a lack of strict randomized controlled designs and inconsistencies in Arjuna preparations being used, the meta-analytic results are not included in our main results section. Nonetheless, the findings from such quasi-randomized studies are still worth mentioning. After analyzing data from 14 arms of 10 different studies enrolling 547 participants, it was observed that Ayurvedic herbal preparations with *Terminalia arjuna* as a main ingredient reduces total cholesterol by 19.47 mg/dL (95% C.I.:30.73, 8.20, *p* = 0.0007), LDL-C by 16.33 mg/dL (95% C.I.:23.21, 9.45, *p* < 0.00001), triglycerides by 11.24 mg/dL (95% C.I.:22.02, 0.46, *p* = 0.04), and raises HDL-C by 5.16 mg/dL (95% C.I.:2.62, 7.69, *p* < 0.00001). Overall, Arjuna may also be considered as a viable herb that can be quite beneficial for patients with hypercholesterolemia. 

#### 4.1.3. Adverse Effects

No serious adverse events were reported in the majority of the studies, except Szapary (2003) [21] reported that one patient each from experimental group and control group had serious side effects and Joseph (2012) reported that one participant in the active control group withdrew due to a five-fold increase in serum creatinine level. Other minor adverse events such as gastrointestinal upset, skin rashes, nausea, and bad odor of breath and body were also reported in few other studies [47,56].

Limitations: Although every effort was made to discover all eligible studies published through December 2020, there is a possibility of some studies still being left behind. Most of the studies that were seemingly eligible did not qualify given our inclusion criteria, but some of the studies did show consistent findings with our study [55,56,57]. A Pakistani study by Zeb et al. [81] examined the impact of garlic powder, coriander powder, and a mixture of the two on lipid profile as compared to a placebo. Garlic powder was found to be the most effective in reducing TC and LDL and increasing HDL, as compared to all other groups. The latest study by Iskander et al. [82] showed that a nutraceutical combination including g gugguluipid showed a significant reduction in TC and LDL levels after 8 weeks of consumption in a randomized, placebo-controlled double-blind trial. Another study by Kuchewar et al. [83] found beneficial effects of Triphala on the lipid profile of patients with dyslipidemia. Many other herbs such as Amla [84,85] have also shown promising results for controlling lipid parameters among patients with hyperlipidemia/dyslipidemia. However, these studies failed to meet the inclusion criteria of the current review. 

## 5. Conclusions

Findings from these systematic reviews and meta-analyses indicate that there is moderate to high strength evidence that several Ayurvedic herbal preparations, i.e., guggulu, garlic, and black cumin, are safe and effective in reducing high levels of cholesterol to a moderate extent. The data suggest that these preparations may be used as first-line therapies or adjuncts to conventional care. We encourage future research to pursue randomized clinical trials with a larger sample size, longer durations, and with clinical outcomes of cardiovascular disease. In addition to the herbs studied, future randomized controlled trials are needed to investigate the efficacy of other Ayurvedic herbal preparations such as Arjuna, Triphala, and Amla in patients with hypercholesterolemia.

## Figures and Tables

**Table 1:** Characteristics of included studies.

Study ID	Intervention & Comparator	Duration of Intervention	Description of Participants	Trial Period	Country, Place	Setting	Ethnic Groups (%)
Prakash 2016 [38]	I: T. arjuna	12 weeks	Age < 20 years, total cholesterol ≥ 200 mg/dL, LDL-C ≥ 130 mg/dL	-	India	Outpatient clinic of university hospital	-
	C: Rosuvastatin						-
Farzaneh 2014 [39]	I: N. sativa	8 weeks	Adult overweight females with sedentary lifestyle and total cholesterol > 200 mg/dL	-	Iran	University clinic	-
	C: Placebo						-
Rathi 2013 [40]	I: Rasonadi leha + Hridroghar churna + Usual care	3 months	Patients of post MI attending private hospital of Betul, Madhya Pradesh, India	-	India	Hospital	-
	C: Usual care						-
Devra 2012 [41]	I: Tulsi extract	3 months	Patients of metabolic syndrome	-	India	Hospital	
	C: Placebo						
Huseini 2012 [42]	I: Aloe	2 months	Patients with type 2 diabetes and hyperlipidemia	-	Iran	Outpatient clinic	-
	C: Placebo						-
Joseph 2012 [43]	I: Amla + Fenugreek	12 weeks	Patients of hypercholesterolemia with total cholesterol > 220 mg/dL	-	-	Outpatient department of tertiary teaching hospital	-
	C: Atorvastatin						-
Sabzghabaee 2012 [44]	I. N. sativa	4 weeks	Patients with toral cholesterol > 200 mg/dL	July 2010–June 2011	Iran	Outpatient clinics of University hospital	-
	C: Placebp						-
Sharma 2012 [45]	I: Lashunadi guggulu	45 days	Clinically diagnosed and confirmed patients of stable angina from out and in-patient departments of two hospitals of Jaipur, India	2002–2004	India	University hospital	-
	C: Placebo						-
Sobenin 2010 [46]	I: Allicor	12 months	Patients with documented CHD, 40–65 years age and s. cholesterol level > 200 mg/dL	-	Russia	Probably research center	-
	C: Placebo						-
Nohr 2009 [47]	I: Guggulu formula	12 (weeks)	Patients from Norwegian general practice who are not taking any prescriptions for hypercholesterolemia, CHD, DM	Feb–May 2003	Oppland and Hedemark counties of Norway	General practice	Native Norwegians
	C: Placebo						Native Norwegians
Qidwai 2009 [48]	I: N. sativa	6 weeks	Patients with total cholesterol level > 180 to 250 mg/dL	Feb 2006–Jan 2007	Pakistan	Outpatient clinics at university hospital	Pakistani
	C: Placebo						Pakistani
Alizadeh-Navaei 2008 [49]	I: Ginger	45 days	Patients of hyperlipidemia with cholesterol > 200 mg/dL or Triglyceride > 200 mg/dL	April 2004–May 2005	Babol, Iran	Cardiac clinic	-
	C: Placebo						-
Sobenin 2008 [50]	I: Allicor	12 weeks	Men with mild hypercholesterolemia	-	Moscow, Russia	Research center	-
	C: Placebo						-
Gardner 2007 a [51]	I: Raw Garlic	6 months	Adults with LDL-C 130–190 mg/dL	Nov 2002–June 2005	USA	University hospital clinic	White (73) Black (4) Asian (18) Hispanic (2)
	C: Placebo						White (64) Asian (14) Hispanic (4)
Gardner 2007 b [51]	I: Garlic in tablets	6 months	Adults with LDL-C 130–190 mg/dL	Nov 2002–June 2005	USA	University hospital clinic	White (66) Black (4) Asian (21) Hispanic (6)
	C: Placebo						White (64) Asian (14) Hispanic (4)
Ashraf 2005 [52]	I: Garlic	12 weeks	Type 2 diabetes mellitus patients with newly diagnosed hyperlipidaemia	-	Karachi, Pakistan	University hospital	-
	C: Placebo						-
Tanamai 2004 [53]	I: Garlic	9 months	Hypercholesterolemia	-	Bangkok, Thailand	Hospital	Thai
	C: Placebo						Thai
Satitvipawee 2003 [54]	I: Garlic	12 weeks	Hypercholesterolemia	-	Thailand	Study center	-
	C: Placebo						-
Szapary 2003 [21]	I: Guggulipid	8 (weeks)	Ambulatory, community-dwelling, healthy adults with hypercholesterolaemia	March 2000–August 2001	Philadelphia, Pa, metropolitan area	University hospital	White (85)
	C: Placebo						White (75)
Venkataramaiah 2002 [55]	I: Abana	8 weeks	Patients with total cholesterol > 200 mg/dL or triglycerides > 200 mg/dL	-	-	-	-
	C: Simvastatin						-
Kannar 2001 [56]	I: Garlic	12 weeks	Volunteers who failed to comply with previous lipid-lowering therapies	-	Victoria, Australia	University clinic	-
	C: Placebo						-
Gardner 2001 [57]	I: Garlic	12 weeks	General public and employees of Stanford University	June–October, 1997	Stanford University, Palo Alto, CA	University hospital	-
	C: Placebo						-
							-
Adler 1997 [58]	I: Garlic	12 weeks	Men with elevated T. cholesterol level > 5.2 mmol/L (200 mg/dL)	-	Guelph, Ontario, Canada	University hospital/clinic	-
	C: Placebo						-
Awasthi 1997 [59]	I: Lashunadi guggulu	2 months	Patients of chronic stbale angina from two hospitals in Jaipur	-	Jaipur, India	University hospital	-
	C: Placebo						-
Gaur 1997 [60]	I: Gugulipid and usual care	4/4 (weeks)	Patients of ischaemic stroke	-	India	-	-
	C: Usual care						-
Singh 1994 [61]	I: Gugguluipid	24 weeks	Patients with hypercholesterolaemia with s. cholesterol level > 200 mg/dL	-	India	-	White (70) Black (30)
	C: Placebo						White (68) Black (32)
Jain 1993 [62]	I: Garlic	12 weeks	Patients with s. total cholesterol level > 220 mg/dL	-	USA	Outptient clinic	-
	C: Placebo						-
Tiwari 1991 [63]	I: Abana	6 months	Diagnosed cases of hypertension and Angina pectoris	-	India	University hospital	-
	C: Propanlol						-
Mader 1990 [64]	I: Garlic	4 months	Patients of hyperlipidaemia from 30 different practices in Germany	-	Germany	General practice	-
	C: Placebo						
Nityanand 1989 [65]	I: Gugguluipid	12 weeks	Patients with s. cholesterol levels > 220 mg/dL	-	India		-
	C: Clofibrate						-
Verma 1988 [66]	I: Guggulu	16 weeks	Patients of hyperlipidaemia between age 40–60 years Type IIa or IIb of Frederichsons classification of hyperlipidemia	-	India	University hospital	-
	C: Placebo						-
Kotiyal 1984 [67]	I: Guggulu	12 weeks	Patients with features of obesity, 10% overweight for one’s height, age, and sex	-	India	Medical OPD of a hospital	-
	C: Placebo						-
Kuppurajan 1978 [68]	I: Guggulu	3 weeks	Patients with s. cholesterol > 300 mg/dL or total lipids > 750 mg/dL	-	India	-	-
	C: Placebo						-

**Table 2:** Risk of bias for included studies.

Study ID	Random Sequence Generation	Allocation Concealment	Blinding	Attrition Bias	Selective Outcome Reporting
Prakash 2016	U	U	U	U	L
Farzaneh 2014	U	U	L	H	L
Rathi 2013	U	U	U	H	L
Devra 2012	U	U	U	U	L
Huseini 2012	L	L	L	L	L
Joseph 2012	U	U	U	U	L
Sabzghabaee 2012	U	L	U	U	L
Sharma 2012	U	U	L	H	L
Sobenin 2010	U	U	L	H	L
Nohr 2009	L	L	L	H	L
Qidwai 2009	U	U	L	H	L
Alizadeh-Navaei 2008	U	U	L	U	L
Sobenin 2008	U	U	L	U	L
Gardner 2007	L	L	L	L	L
Ashraf 2005	U	U	H	L	L
Tanamai 2004	U	U	L	H	L
Satitvipawee 2003	L	L	L	L	L
Szapary 2003	L	L	L	L	L
Venkataramaiah 2002	U	U	U	U	U
Kannar 2001	U	U	L	L	L
Gardner 2001	U	U	L	L	L
Adler 1997	U	U	L	L	L
Awasthi 1997	U	U	L	H	L
Gaur 1997	U	U	U	H	L
Singh 1994	U	U	L	H	L
Jain 1993	U	U	H	L	L
Tiwari 1991	U	U	L	H	L
Mader 1990	L	L	L	L	L
Nityanand 1989	U	U	L	H	L
Verma 1988	U	U	L	U	L
Kotiyal 1984	U	U	L	U	L
Kuppurajan 1978	U	U	L	H	L

L: low risk; H: high risk; U: unclear risk.

**Table 3:** Effect of Ayurvedic herbal preparations in total cholesterol (mg/dL).

Outcome or Subgroup	Studies	Participants	Statistical Method	Effect Estimate
1.1 Total Cholesterol level	24	1386	Mean Difference (IV, Random, 95% CI)	
1.1.1 Garlic	11	813	Mean Difference (IV, Random, 95% CI)	−12.45 (−18.68, −6.22)
1.1.2 Guggulu	8	380	Mean Difference (IV, Random, 95% CI)	−16.78 (−30.96, −2.61)
1.1.3 Nigella	3	163	Mean Difference (IV, Random, 95% CI)	−9.28 (−17.36, −1.19)
1.1.5 Garlic + guggulu	2	30	Mean Difference (IV, Random, 95% CI)	−38.28 (−55.11, −21.44)

**Table 4:** Effect of Ayurvedic herbal preparations on LDL-C (mg/dL).

Outcome or Subgroup	Studies	Participants	Statistical Method	Effect Estimate
1.2 LDL-Cholesterol level	21	1183	Mean Difference (IV, Random, 95% CI)	
1.2.1 Garlic	12	734	Mean Difference (IV, Random, 95% CI)	−10.37 (−17.58, −3.16)
1.2.2 Guggulu	5	266	Mean Difference (IV, Random, 95% CI)	−18.78 (−34.07, −3.48)
1.2.3 Nigella	3	163	Mean Difference (IV, Random, 95% CI)	−2.12 (−7.85, 3.60)
1.2.5 Garlic + guggulu	1	20	Mean Difference (IV, Random, 95% CI)	−51.43 (−69.87, −32.99)

**Table 5:** Effect of Ayurvedic herbal preparations on triglycerides (mg/dL).

Outcome or Subgroup	Studies	Participants	Statistical Method	Effect Estimate
1.3 Triglycerides level	23	1364	Mean Difference (IV, Random, 95% CI)	
1.3.1 Garlic	12	819	Mean Difference (IV, Random, 95% CI)	−3.10 (−16.63, 10.42)
1.3.2 Guggulu	6	352	Mean Difference (IV, Random, 95% CI)	−7.35 (−23.29, 8.59)
1.3.3 Nigella	3	163	Mean Difference (IV, Random, 95% CI)	−21.09 (−44.96, 2.77)
1.3.5 Garlic + guggulu	2	30	Mean Difference (IV, Random, 95% CI)	−13.23 (−28.53, 2.07)

**Table 6:** Effect of Ayurvedic herbal preparations on HDL-C (mg/dL).

Outcome or Subgroup	Studies	Participants	Statistical Method	Effect Estimate
1.4 HDL-Cholesterol level	21	1186	Mean Difference (IV, Random, 95% CI)	
1.4.1 Garlic	12	736	Mean Difference (IV, Random, 95% CI)	−2.91 (−9.19, 3.37)
1.4.2 Guggulu	5	267	Mean Difference (IV, Random, 95% CI)	2.19 (0.27, 4.12)
1.4.3 Nigella	3	163	Mean Difference (IV, Random, 95% CI)	1.92 (−1.62, 5.45)
1.4.5 Garlic + guggulu	1	20	Mean Difference (IV, Random, 95% CI)	10.00 (5.87, 14.13)

## References

[1] Mendis S., Puska P., Norrving B. (2011). Global Atlas on Cardiovascular Disease Prevention and Control.

[2] Centers for Disease Control and Prevention (2011). Vital signs: Prevalence, treatment, and control of high levels of low-density lipoprotein cholesterol-United States, 1999–2002 and 2005–2008. MMWR.

[3] Gupta A., Sehgal V., Mehan S. (2011). Hyperlipidemia: An updated review. Int. J. Biopharm. Toxicol. Res..

[4] National Cholesterol Education Program (NCEP) (2002). Expert Panel on Detection, Evaluation, and Treatment of High Blood Cholesterol in Adults (Adult Treatment Panel III) Final Report. Circulation.

[5] (1984). The Lipid Research Clinics Coronary Primary Prevention Trial Results. JAMA.

[6] (1984). The Lipid Research Clinics Coronary Primary Prevention Trial Results. II. The Relationship of Reduction in Incidence of Coronary Heart Disease to Cholesterol Lowering. JAMA.

[7] Punekar R.S., Fox K.M., Richhariya A., Fisher M.D., Cziraky M., Gandra S.R., Toth P.P. (2015). Burden of First and Recurrent Cardiovascular Events among Patients with Hyperlipidemia. Clin. Cardiol..

[8] Bahia L.R., Rosa R.S., Santos R.D., Araújo D.V. (2018). Estimated costs of hospitalization due to coronary artery disease attributable to familial hypercholesterolemia in the Brazilian public health system. Arch. Endocrinol. Metab..

[9] Soni A. Top 10 Most Costly Conditions among Men and Women, 2008: Estimates for the U.S. Civilian Noninstitutionalized Adult Population, Age 18 and Older, Med. Expend. Panel Surv. (2011) Statistical Brief #331). https://meps.ahrq.gov/data_files/publications/st331/stat331.shtml.

[10] Scirica B.M., Cannon C.P. (2005). Treatment of Elevated Cholesterol. Circulation.

[11] Golomb B.A., Evans M.A. (2008). Statin Adverse Effects. Am. J. Cardiovasc. Drugs.

[12] Chrysant S.G. (2017). New onset diabetes mellitus induced by statins: Current evidence. Postgrad. Med..

[13] Bove M., Cicero A.F., Borghi C. (2019). Emerging drugs for the treatment of hypercholesterolemia. Expert Opin. Emerg. Drugs.

[14] Arnett D.K., Jacobs D.R., Luepker R.V., Blackburn H., Armstrong C., Claas S.A. (2005). Twenty-Year Trends in Serum Cholesterol, Hypercholesterolemia, and Cholesterol Medication Use: The Minnesota Heart Survey. Circulation.

[15] Qidwai W., Jahan F., Nanji K. (2014). Role of Complementary and Alternative Medicine in Controlling Dyslipidemia. Evid. Based Complement. Altern. Med..

[16] Singh B.B., Vinjamury S.P., Der-Martirosian C., Kubik E., Mishra L.C., Shepard N.P., Singh V.J., Meier M., Madhu S.G. (2007). Ayurvedic and collateral herbal treatments for hyperlipidemia: A systematic review of randomized controlled trials and qua-si-experimental designs. Altern. Ther. Health Med..

[17] Patwardhan B. (2014). Bridging Ayurveda with evidence-based scientific approaches in medicine. EPMA J..

[18] Kessler C., Wischnewsky M., Michalsen A., Eisenmann C., Melzer J. (2013). Ayurveda: Between Religion, Spirituality, and Medicine. Evid. Based Complement. Altern. Med..

[19] Gupta R., Singhal S., Goyle A., Sharma V.N. (2001). Antioxidant and hypocholesterolaemic effects of Terminalia arjuna tree-bark powder: A randomised placebo-controlled trial. J. Assoc. Physicians India.

[20] Silagy C., Neil A. (1994). Garlic as a Lipid Lowering Agent—A Meta-Analysis. J. R. Coll. Physicians Lond..

[21] Szapary P.O., Wolfe M.L., Bloedon L.T., Cucchiara A.J., DerMarderosian A.H., Cirigliano M.D., Rader D.J. (2003). Guggulipid for the Treatment of Hypercholesterolemia. JAMA.

[22] Qidwai W., Yeoh P.N., Inem V., Nanji K., Ashfaq T. (2013). Role of Complementary and Alternative Medicine in Cardiovascular Diseases. Evid. Based Complement. Altern. Med..

[23] Patwardhan K. (2019). Promoting evidence-base for Ayurveda. J. Ayurveda Integr. Med..

[24] Dwivedi S. (2007). Terminalia arjuna Wight & Arn.—A useful drug for cardiovascular disorders. J. Ethnopharmacol..

[25] Kaur N., Shafiq N., Negi H., Pandey A., Reddy S., Kaur H., Chadha N., Malhotra S. (2014). Terminalia arjunain Chronic Stable Angina: Systematic Review and Meta-Analysis. Cardiol. Res. Pract..

[26] Ulbricht C., Basch E., Szapary P., Hammerness P., Axentsev S., Boon H., Kroll D., Garraway L., Vora M., Woods J. (2005). Guggul for hyperlipidemia: A review by the Natural Standard Research Collaboration. Complement. Ther. Med..

[27] Ahmada A., Husainb A., Mujeebc M., Alam Khan S., Najmi A.K., Siddique N.A., Damanhouri Z.A., Anwarh F. (2013). A review on therapeutic potential of Nigella sativa: A miracle herb. Asian Pac. J. Trop. Biomed..

[28] Liberati A., Altman D.G., Tetzlaff J., Mulrow C., Gøtzsche P.C., Ioannidis J.P.A., Clarke M., Devereaux P.J., Kleijnen J., Moher D. (2009). The PRISMA statement for reporting systematic reviews and meta-analyses of studies that evaluate health care interventions: Explanation and elaboration. PLoS Med..

[29] Higgins J.P.T., Green S. Cochrane Handbook for Systematic Reviews of Interventions, Version 5.1.0. Updated March 2011. The Cochrane Collaboration, 2001. www.cochrane-handbook.org.

[30] Gyawali D., Schneider R.H., Orme-Johnson D.W., Ramaratnam S. (2016). Ayurvedic herbal preparations for hypercholesterolaemia. Cochrane Database Syst. Rev..

[31] Deeks J., Higgins J. Statistical algorithms in Review Manager 5. https://training.cochrane.org/handbook/statistical-methods-revman5.

[32] Higgins J.P.T., Altman D.G., Gøtzsche P.C., Jüni P., Moher D., Oxman A.D., Savović J., Schulz K.F., Weeks L., Sterne J.A.C. (2011). The Cochrane Collaboration’s tool for assessing risk of bias in randomised trials. BMJ.

[33] Higgins J.P.T., Thompson S.G., Deeks J.J., Altman D.G. (2003). Measuring inconsistency in meta-analyses. BMJ.

[34] Sterne J., Sutton A.J., Ioannidis J.P.A., Terrin N., Jones D.R., Lau J., Carpenter J., Rücker G., Harbord R.M., Schmid C.H. (2011). Recommendations for examining and interpreting funnel plot asymmetry in meta-analyses of randomised controlled trials. BMJ.

[35] Wood L., Egger M., Gluud L.L., Schulz K.F., Jüni P., Altman D.G., Gluud C., Martin R.M., Wood A.J.G., Sterne J.A.C. (2008). Empirical evidence of bias in treatment effect estimates in controlled trials with different interventions and outcomes: Meta-epidemiological study. BMJ.

[36] Higgins J.P.T., Thompson S.G., Spiegelhalter D.J. (2009). A re-evaluation of random-effects meta-analysis. J. R. Stat. Soc. Ser. A.

[37] Riley R.D., Higgins J.P.T., Deeks J.J. (2011). Interpretation of random effects meta-analyses. BMJ.

[38] Prakash V., Sehgal V., Bajaj V., Singh H. (2016). To compare the effects of Terminalia Arjuna with Rosuvastatin on total cholesterol and low-density lipoprotein cholesterol. Int. J. Med. Dent. Sci..

[39] Farzaneh E., Nia F.R., Mehrtash M., Mirmoeini F.S., Jalilvand M. (2014). The effects of 8-week Nigella sativa supplementation and aerobic training on lipid profile and VO_2_ max in sedentary overweight females. Int. J. Prev. Med..

[40] Rathi R., Rathi B.J. (2013). A clinical comparative study of rasonadi leha with hridrogadi churna in coronary artery disease. Punarnav.

[41] Devra D.K., Mathur K.C., Agrawal R.P., Bhadu I., Goyal S., Agarwal V. (2012). Effect of Tulsi (*Ocimum sanctum* Linn.) on clinical and biochemical parameters of metabolic syndrome. J. Nat. Remed..

[42] Huseini H.F., Kianbakht S., Hajiaghaee R., Dabaghian F.H. (2012). Anti-hyperglycemic and anti-hypercholesterolemic effects of Aloe vera leaf gel in hyperlipidemic type 2 diabetic patients: A randomized double-blind placebo-controlled clinical trial. Planta Med..

[43] Joseph S., Santhosh D., Udupa A.L., Gupta S., Ojeh N., Rathnakar U.P., Benegal D., Benegal A., Shubha H.V., Rao S.P. (2012). Hypolipidemic Activity of Phyllanthus Emblica *Linn* (Amla) & Trigonella Foenum Graecum (Fenugreek) Combination In Hypercholesterolemic Subjects–A Prospective, Randomised, Parallel, Open-Label, Positive Controlled Study. Asian J. Bio. Pharma. Res..

[44] Sabzghabaee A.M., Dianatkhah M., Sarrafzadegan N., Asgary S., Ghannadi A. (2012). Clinical evaluation of Nigella sativa seeds for the treatment of hyperlipidemia: A randomized, placebo controlled clinical trial. Med. Arch..

[45] Sharma A.K., Sharma S.M., Sharma S.P., Sharma A.K. (2012). Management of stable angina with lashunadi guggulu- an ayurvedic formulation. Ann. Ayurvedic. Med..

[46] Sobenin I.A., Pryanishnikov V.V., Kunnova L.M., Rabinovich Y.A., Martirosyan D.M., Orekhov A.N. (2010). The effects of time-released garlic powder tablets on multifunctional cardiovascular risk in patients with coronary artery disease. Lipids Health Dis..

[47] Nohr L.A., Rasmussen L.B., Straand J. (2009). Resin from the mukul myrrh tree, guggul, can it be used for treating hypercholesterolemia? A randomized, controlled study. Complement. Ther. Med..

[48] Qidwai W., Bin Hamza H., Qureshi R., Gilani A. (2009). Effectiveness, safety, and tolerability of powdered nigella sativa (kalonji) seed in capsules on serum lipid levels, blood sugar, blood pressure, and body weight in adults: Results of a randomized, double-blind controlled trial. J. Altern. Complement. Med..

[49] Alizadeh-Navaei R., Roozbeh F., Saravi M., Pouramir M., Jalali F., Moghadamnia A.A. (2008). Investigation of the effect of ginger on the lipid levels. A double blind controlled clinical trial. Saudi Med. J..

[50] Sobenin I.A., Andrianova I.V., Demidova O.N., Gorchakova T., Orekhov A.N. (2008). Lipid-lowering effects of time-released garlic powder tablets in double-blinded placebo-controlled randomized study. J. Ather. Thromb..

[51] Gardner C.D., Lawson L.D., Block E., Chatterjee L.M., Kiazand A., Balise R.R., Kraemer H.C. (2007). Effect of raw garlic vs commercial garlic supplements on plasma lipid concentrations in adults with moderate hypercholesterolemia. Arch. Intern. Med..

[52] Ashraf R., Aamir K., Shaikh A.R., Ahmed T. (2005). Effects of garlic on dyslipidemia in patients with type 2 diabetes mellitus. J. Ayub. Med. Coll. Abbottabad..

[53] Tanamai J., Veeramanomai S., Indrakosas N. (2004). The efficacy of cholesterol-lowering action and side effects of garlic enteric coated tablets in man. J. Med. Ass. Thailand.

[54] Satitvipawee P., Suparp J., Podhipak A., Viwatwongkasem C. (2003). Can garlic extract supplement lower blood pressure in hypercholesterolemic subjects?. J. Public Health.

[55] Double-Blind Comparative Clinical Trial of Abana and Simvastatin in Hyperlipidaemia. https://cdn.greensoft.mn/uploads/users/1977/files/tsakhurtumur%20sudalga/Abana%20sudalgaa.pdf.

[56] Kannar D., Wattanapenpaiboon N., Savige G.S., Wahlqvist M.L. (2001). Hypocholesterolemic effect of an enteric-coated garlic supplement. J. Am. Coll. Nutr..

[57] Gardner C.D., Chatterjee L.M., Carlson J.J. (2001). The effect of a garlic preparation on plasma lipid levels in moderately hypercholesterolemic adults. Atherosclerosis.

[58] Adler A.J., Holub B.J. (1997). Effect of garlic and fish-oil supplementation on serum lipid and lipoprotein concentrations in hypercholesterolemic men. Am. J. Clin. Nutr..

[59] Awasthi A., Kothari K., Sharma R. (1997). Evaluation of the effect of the indigenous herbal drug lashunadi guggulu in management of chronic stable Angina. Aryavaidyan Xi.

[60] Gaur S.P., Garg R.K., Kar A.M. (1997). Gugulipid, a new hypolipidaemic agent, in patients of acute ischaemic stroke: Effect on clinical outcome, platelet function and serum lipids. Asia Pacif. J. Pharm..

[61] Singh R.B., Niaz M.A., Ghosh S. (1994). Hypolipidemic and antioxidant effects of Commiphora mukul as an adjunct to dietary therapy in patients with hypercholesterolemia. Cardiovasc. Drugs. Ther..

[62] Jain A.K., Vargas R., Gotzkowsky S., McMahon F.G. (1993). Can garlic reduce levels of serum lipids? A controlled clinical study. Am. J. Med..

[63] Tiwari A.K., Agrawal A., Gode J.D., Dubey G.P. (1991). Perspective, randomised crossover study of propranolol and Abana in hypertensive patients: Effect on lipids and lipoproteins. Antiseptic..

[64] Mader F.H. (1990). Treatment of hyperlipidaemia with garlic-powder tablets. Evidence from the German Association of General Practitioners’ multicentric placebo-controlled double-blind study. Arzneimittel-Forschung.

[65] Nityanand S., Srivastava J.S., Asthana O.P. (1989). Clinical trials with gugulipid. A new hypolipidaemic agent. J. Assoc. Physicians Ind..

[66] Verma S.K., Bordia A. (1988). Effect of Commiphora mukul (gum guggulu) in patients of hyperlipidemia with special reference to HDL-cholesterol. Indian J. Med. Res..

[67] Kotiyal J.P., Singh D.S., Bisht D.B. (1985). Gum guggulu (Commiphora mukul) fraction A in obesity—A double-blind clinical trial. J. Res. Ayur. Siddha..

[68] Kuppurajan K., Rajagopalan S.S., Rao T.K., Sitaraman R. (1978). Effect of guggul (Commiphora mukul–Engl.) on serum lipids in obese, hypercholesterolemic and hyperlipemic cases. J. Assoc. Physicians India..

[69] Law M.R., Wald N.J., Thompson S.G. (1994). By how much and how quickly does reduction in serum cholesterol concentration lower risk of ischaemic heart disease?. BMJ.

[70] Delahoy P.J., Magliano D.J., Webb K., Grobler M., Liew D. (2009). The relationship between reduction in low-density lipoprotein cholesterol by statins and reduction in risk of cardiovascular outcomes: An updated meta-analysis. Clin. Ther..

[71] Chunekar K.C. (2009). Bhavprakash Nighantu of Bhavmisra.

[72] Urizar N.L., Moore D.D. (2003). GUGULIPID: A Natural Cholesterol-Lowering Agent. Annu. Rev. Nutr..

[73] Golomb B.A. (2015). Misinterpretation of trial evidence on statin adverse effects may harm patients. Eur. J. Prev. Cardiol..

[74] Ried K., Toben C., Fakler P. (2013). Effect of garlic on serum lipids: An updated meta-analysis. Nutr. Rev..

[75] Reinhart K.M., Talati R., White C.M., Coleman C.I. (2009). The impact of garlic on lipid parameters: A systematic review and meta-analysis. Nutr. Res. Rev..

[76] Stevinson C., Pittler M., Ernst E. (2001). Garlic for treating hypercholesterolemia. ACC Curr. J. Rev..

[77] Amagase H., Petesch B.L., Matsuura H., Kasuga S., Itakura Y. (2001). Intake of garlic and its bioactive components. J. Nutr..

[78] Shastri K. (2001). Sushruta Samhita of Maharshi Sushruta Part I.

[79] Wang H.-P., Yang J., Qin L.-Q., Yang X.-J. (2015). Effect of Garlic on Blood Pressure: A Meta-Analysis. J. Clin. Hypertens..

[80] Sahebkar A., Beccuti G., Simental-Mendía L.E., Nobili V., Bo S. (2016). Nigella sativa (black seed) effects on plasma lipid concentrations in humans: A systematic review and meta-analysis of randomized placebo-controlled trials. Pharmacol. Res..

[81] Zeb F., Safdar M., Fatima S., Khan S., Alam S., Muhammad M., Syed A., Habib F., Shakoor H. (2018). Supplementation of garlic and coriander seed powder: Impact on body mass index, lipid profile and blood pressure of hyperlipidemic patients. Pak. J. Pharm. Sci..

[82] Iskandar I., Harahap Y., Wijayanti T.R., Sandra M., Prasaja B., Cahyaningsih P., Rahayu T. (2020). Efficacy and tolerability of a nutraceutical combination of red yeast rice, guggulipid, and chromium picolinate evaluated in a randomized, placebo-controlled, double-blind study. Complement. Ther. Med..

[83] Kuchewar V.V. (2017). Efficacy and safety study of triphala in patients of dyslipidemia: A pilot project. Int. J. Res. Ayurveda Pharm..

[84] Bhatt J., Hemavathi K.G., Gopa B. (2012). A comparative clinical study of hypolipidemic efficacy of Amla (Emblica officinalis) with 3-hydroxy-3-methylglutaryl-coenzyme-A reductase inhibitor simvastatin. Indian J. Pharmacol..

[85] Upadya H., Prabhu S., Prasad A., Subramanian D., Gupta S., Goel A. (2019). A randomized, double blind, placebo controlled, multicenter clinical trial to assess the efficacy and safety of Emblica officinalis extract in patients with dyslipidemia. BMC Complement. Altern. Med..

